# A Framework for Navigating Institutional Review Board (IRB) Oversight in the Complicated Zone of Research

**DOI:** 10.7759/cureus.844

**Published:** 2016-10-25

**Authors:** Gretchen E Parker

**Affiliations:** 1 Ethics Review, Pearl IRB LLC

**Keywords:** institutional review board, clinical research, efficiency, ethics committee, biomedical research, human subjects research, research ethics

## Abstract

The treatment therapies and technologies currently emerging from the rapidly evolving health care industry must undergo full examination in a clinical setting if they are to be marketed to the public. All elements of clinical studies involving human subjects must undergo thorough IRB review before study activities can commence. Regulations regarding IRB oversight apply to all clinical studies—including retrospective examinations of private medical data and identifiable biological samples. It is not uncommon for researchers to be unsure whether, or on what level, IRB review and oversight are required for a particular project. Yet, if human subjects or their private medical data are utilized in a study, peer-reviewed journals will require relevant IRB approval information be provided as a requirement for publication. This article examines IRB processes and review types, offers insight into the IRB decision-making process, and emphasizes the importance of engaging an IRB consultant early in the clinical study design process.

## Introduction

From Phase I through post-market analysis, clinical investigations are continually developed and implemented to test a product or intervention’s safety and effectiveness. The assessment of interventions related to human subjects typically begins by performing preclinical animal tests to determine how the drug and/or intervention works and if it will be safe enough to be tested in humans. Preclinical studies also assess product absorption, excretion, and metabolism, and aid in identifying the correct human dosage. A series of clinical trials in three phases is then performed to test if the product is effective and safe in humans. A fourth phase, post-market analysis, occurs following regulatory approval of the product. Safety and effectiveness data must be generated and assessed throughout a product’s total life cycle.

The Department of Health and Human Services (DHHS) Office for Human Research Protections (OHRP) and the Food and Drug Administration (FDA) are two agencies regulating clinical investigations. When implementing an investigational protocol, a clinical study’s principal investigator (PI) is responsible for following and complying with all applicable federal regulations regarding human subject research [1]. These responsibilities include obtaining initial and continuing IRB review and approval of the proposed research study. This obligation is especially important if you want to use these data for an investigational product application or in a publication. IRBs were formed as a result of disasters occurring in the field of human research. Studies including the Milgram obedience study, the Willowbrook hepatitis experiments, the Jewish Chronic Disease Hospital study, the Public Health Service (Tuskegee) syphilis study, and the Nazi medical experiments are all examples where researchers severely mistreated study subjects [2]. In all these examples, the subjects were not offered the same considerations and protections as other populations. In fact, they would have been better off had they never been included in the studies.

The IRB has the power to approve, require modifications to (in order to secure approval), or disapprove human subjects research. An IRB must review and approve all research involving humans before the study begins. An IRB may not review projects retrospectively. This includes proposed research involving previously collected human fluid and tissue samples and existing data, as well as advertising and recruitment procedures. In order to approve research, an IRB must determine that risks to subjects are minimized and reasonable in relation to anticipated benefits, selection of subjects is equitable, subjects are adequately consented, data are appropriately monitored, the privacy of subjects is maintained and the resulting data kept confidential, and vulnerable subjects are protected [3]. In short, the objective of an IRB is to ensure, both in advance and by periodic review and monitoring, that the rights and welfare of humans participating as subjects in a research study are protected. To accomplish this goal, IRBs meet to assess research protocols and their associated materials (e.g., informed consent documents and advertisements). IRB oversight is complex and includes a number of activities with numerous levels of IRB review and supervision.

## Technical report

Unfortunately, instances where clinical investigators fail to acquire proper IRB oversight have occurred. In these cases, the investigator is often dismayed to find out an IRB cannot retrospectively review their study and, because of such, they cannot publish results in a peer-reviewed journal or use the data in a regulatory submission. Questions that arise often include:

*- *Does an IRB need to oversee my research study?

- Will my project require continual IRB oversight?

- What level of IRB review does my investigation require?

- Do I need to let the IRB know when my project is done?

There are a number of questions that an IRB reviewer must consider before these issues can be resolved. When determining the appropriate level of IRB oversight, an IRB reviewer must ask four basic questions about the study:

1. Is it research/are you engaged in research?

2. If yes, does the research involve human subjects?

3. If yes, is your research exempt?

4. If no, what level of IRB review is appropriate?

### Is it research? What is a human subject? Are you engaged in research?

Definitions often differ between the FDA and DHHS/OHRP. Definitions of "research" are shown in Table 1. “Human subject” is defined in Table 2. If you believe your research meets the federal definition for human subjects research you must apply for IRB review and approval of your study prior to any commencement of intervention.

“Engaged in research” means that employees (or agents of an investigator or institution) are intervening or interacting with human subjects for research purposes or to obtain individually identifiable private health information for research purposes [4-5]. In brief, if your systematic investigation designed to develop or contribute to generalizable knowledge also interacts with human subjects or their private health information, you will need IRB approval and oversight. For example, you are considered “engaged in human subjects research” if you:

- Manipulate a human subject’s environment for research purposes (e.g., controlling environmental temperature, sound, or light; and coordinating social interactions).

- Interact with human subjects by participating in protocol-specified communication or personal contact (e.g., accruing specimens and administering questionnaires).

- Use invasive or noninvasive procedures for research purposes with human subjects.

- Are responsible for the informed consent process.

- Obtain private health information or identifiable biological specimens from any source for research purposes. In this case, one does not necessarily need to have direct interaction or intervention with the study subject.

- Receive an award (e.g., a grant or contract) directly from DHHS for non-exempt human subjects research. This is the case even if all research activities are carried out by another institution or individual.

For many clinical investigations, the role of some investigative team members might not meet the definition of being engaged in research. For example, if a technician receives a de-identified tissue specimen and they have no way to re-identify the tissue donor, the technician is not engaged in research. Therefore, under some circumstances, certain individuals may not be considered engaged in research even though study investigators elsewhere in their organization are.

In one example, de-identified tissue samples are sent to a laboratory for analysis. If laboratory personnel do not enroll subjects in the study, then the lab is not engaged in human subjects research. There is one exception: when the study is sponsored by DHHS and the PI is also from the lab performing the analysis, an IRB is required to review the study [6]. This is the case even when the PI is not personally engaged in the conduct of the study. DHHS rationalization for this is that the PI is ultimately responsible for oversight of the human subjects research being conducted at the site.

**Table 1:** Definitions of Research

Agency	Regulation	Definition
OHRP	[45 CFR 46.102(d)]	Research: "A systematic investigation designed to develop or contribute to generalizable knowledge."
FDA	[21 CFR 56.10]	Clinical Investigation: "Involves use of a test article (i.e., drug, device, food substance or biologic), one or more human subjects, meets requirements for prior submission to FDA, or results are intended to be part of an application for research or marketing permit."

**Table 2:** Definitions of Human Subject

Agency	Regulation	Definition
OHRP	[45 CFR 46.102(f)]	Human subject (DHHS): A living individual about whom an investigator (whether faculty, student, or staff) conducting research obtains: (1) data through intervention or interaction with the individual; or (2) identifiable private information.
FDA	[21 CFR 56.102(e)] (Drug, Food, Biologic)	Human subject (FDA): "An individual who is or becomes a participant in research, either as a recipient of the test article or as a control. A subject may be either a healthy individual or a patient.”
FDA	[21 CFR 812.3(p)] (Medical Devices Only)	Human subject (FDA for medical devices): "A human who participates in an investigation, either as an individual on whom or on whose specimen an investigational device is used or as a control. A subject may be in normal health or may have a medical condition or disease.” NOTE: This definition includes use of tissue specimens even if they are unidentified.

### What types of studies might be considered exempt?

Research can be determined to be “exempt” if it is no more than “minimal risk” per 45 CFR §46.405 and fits one of the six federally designated exempt review categories listed in 45 CFR §46.101. Under this specific statute, studies that may qualify for exempt must still be submitted to the IRB for an exemption determination review prior to any intervention.

The six categories of human subjects research that is exempt from IRB oversight are:

1. Research conducted in established or commonly accepted educational settings, involving standard educational practices.

2. Research necessitating the use of educational tests, interview or survey procedures, or assessment of public behavior.

3. Research involving the use of educational tests, interview or survey procedures, or observation of public behavior when the human subjects are elected or appointed public officials or candidate for public office.

4. Research involving the collection or study of existing data, documents, records, pathological specimens, or diagnostic specimens. This category applies only if these sources are publicly available or if the information is recorded by the investigator in such a way that the subject cannot be identified directly or through identifiers linked to the subject.

5. Research and demonstration projects which are conducted by, or subject to the approval of, department or agency heads, and which are designed to examine public benefit or service programs.

6. Taste and food quality evaluation and consumer acceptance studies.

But what if samples contain identifying donor information (the inverse of #4 above)? What level of review would be appropriate then? Expedited or full board? Since this is a minimal risk study, it could be expedited. However, if the study is greater than minimal risk (e.g., the investigator is prospectively collecting samples via invasive means) the study would need to go before the full board for review. These two types of review are discussed below.

### What is an expedited review?

Expedited review allows the IRB chairperson or another experienced IRB reviewer designated by the chairperson to evaluate and approve minimal risk research. Reviewers conducting an expedited review may exercise all of the authority of the IRB except that they may not reject a study. When a reviewer cannot approve the research under expedited review, the study is referred to the full board for review.

To qualify for an expedited review, research must fall into one of nine federally defined, expedited categories [7]. These categories involve collection of non-anonymous samples and data in a manner that involves no more than minimal risk to subjects. Expedited categories include:

1. Clinical studies of drugs and medical devices only when certain conditions are met.

2. Collection of blood samples by heel/finger stick or venipuncture in certain patient populations and within certain amounts.

3. Prospective collection of biological specimens for research purposes by noninvasive means (e.g., placenta removed at delivery and nail clippings).

4. Collection of data through noninvasive procedures (not involving general anesthesia or sedation) routinely employed in clinical practice, excluding procedures involving X-rays or microwaves (e.g., magnetic resonance imaging and ultrasound).

5. Research involving materials that have been collected, or will be collected, solely for non-research purposes.

6. Collection of data from voice, video, digital, or image recordings made for research purposes.

7. Research on individual or group characteristics or behavior; or research employing survey, interview, oral history, focus group, program evaluation, human factors evaluation, or quality assurance methods.

8 and 9. Certain continuing reviews.

Some examples of expedited research are:

- Studies involving the prospective collection of hair or saliva samples.

- Collection of blood samples from healthy volunteers.

- Studies of existing pathological specimens with patient identifiers.

### What is a full board review?

A full board review is necessary for any proposed human subject research that does not fall into either the exempt or expedited review categories. This research is deemed greater than minimal risk. Some examples of this type of research are:

- Studies involving the use of a drug for which an investigational new drug (IND) application is required.

- Studies involving vulnerable subjects. These may include children, pregnant women, prisoners, or employees.

- Studies involving the prospective collection of tissue samples via invasive means.

- Research involving general anesthesia or sedation.

- Some modifications to research initially approved by a convened IRB.

**Figure 1:**
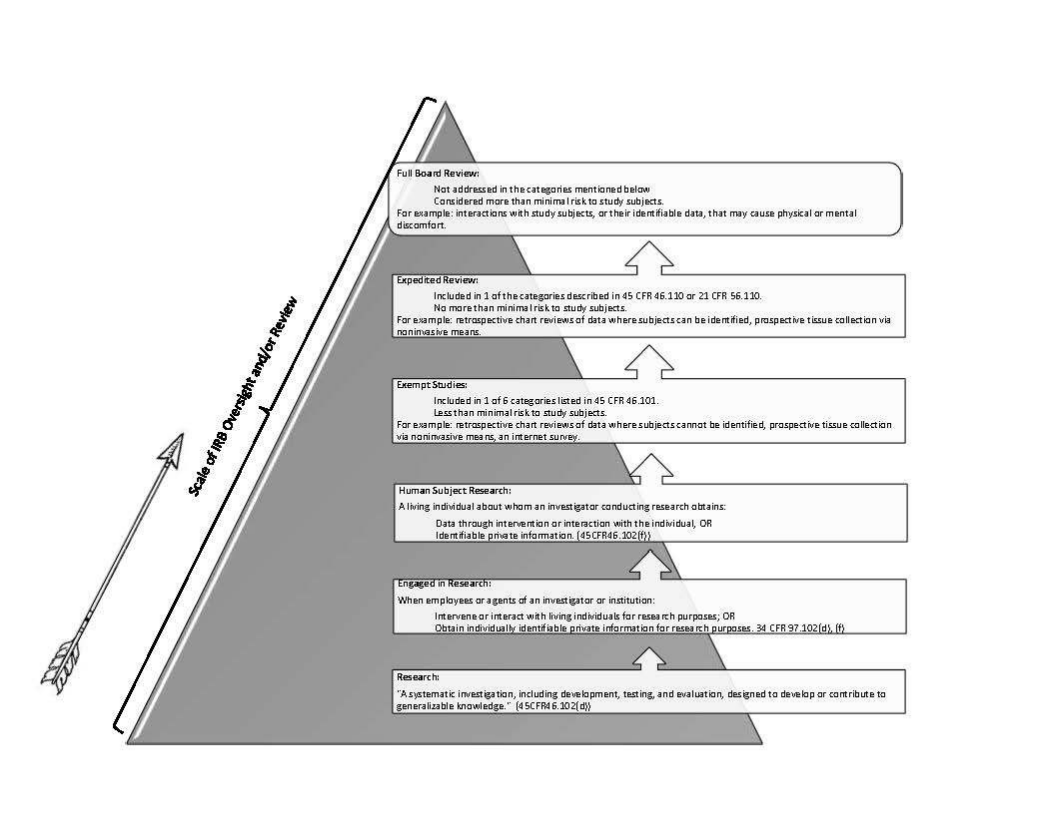
Levels of IRB Oversight and Review

**Figure 2:**
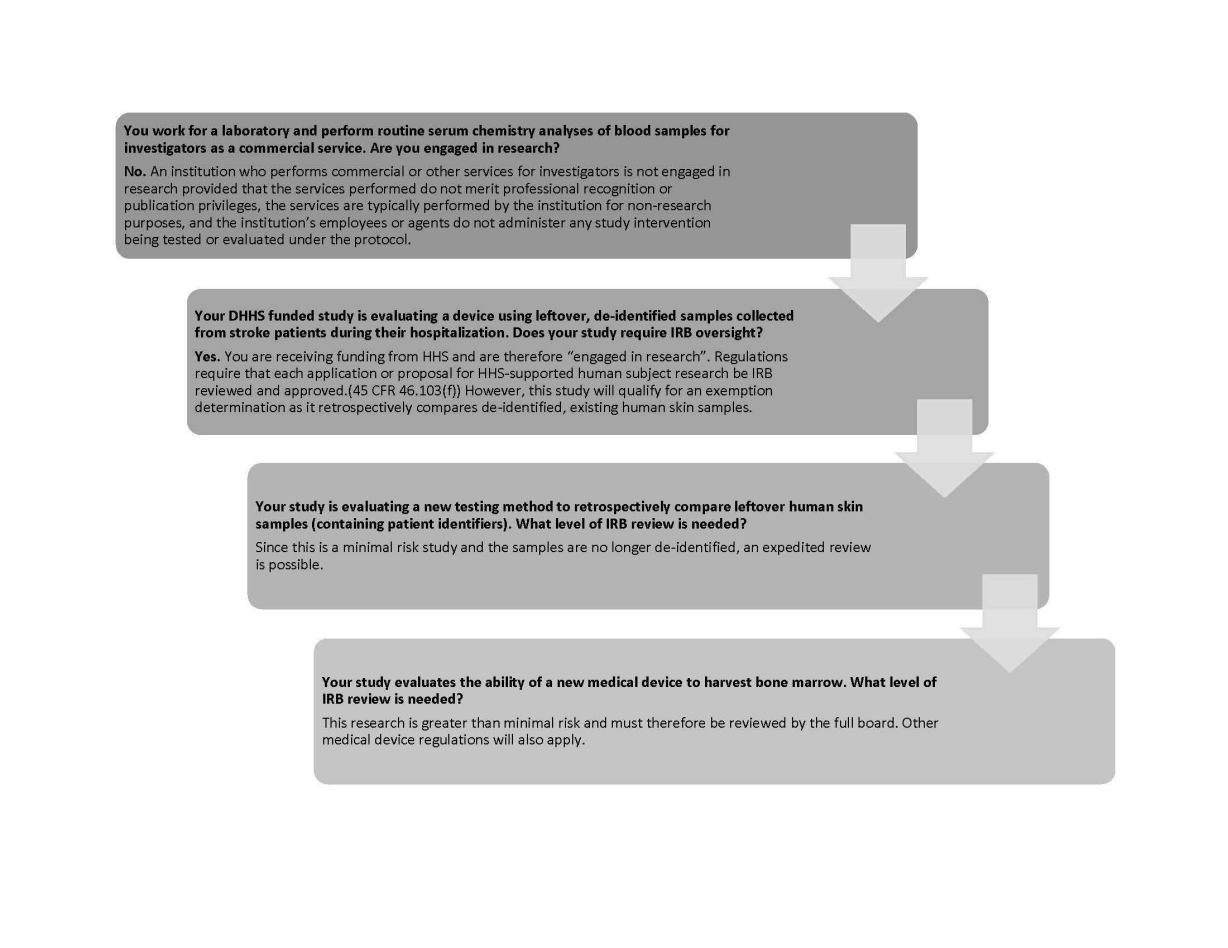
Oversight Scenarios

Once a PI has supplied an initial assessment, an IRB will verify or correct the level of review needed for a study before rendering a decision and forwarding approval documentation. Figure 1 summarizes various levels of IRB oversight and Figure 2 gives examples of situations which may be presented to an IRB for clarification.

### Are changes to the regulations coming?

The short answer is yes. Changes are on the way. What is unclear is what will be changing or when the changes will take place. As a result of the technological advances currently impacting the clinical research industry, the DHHS has proposed changes to the Common Rule (45 CFR § 46) which could result in a major overhaul of human research regulatory requirements [8]. The DHHS proposed nineteen changes to the Common Rule; however, five major areas impacting clinical research could be affected. Briefly, these include: 

1. The harmonization of OHRP and FDA guidelines.

2. The simplification of informed consent requirements and forms.

3. Clinical research regulation changes: Research in which the subjects may be placed at risk and are identifiable could qualify for an exemption determination, thereby expanding the exempt category. Additionally, the existing six categories of research that are considered exempt may have new guidelines to follow.

4. Research involving biobanks: At present, residual biospecimens can be utilized for research purposes without individual consent by stripping the specimens of identifiers. The Notice of Proposed Rulemaking (NPRM) proposes written consent for research of this nature.

5. Changes to IRB scope: Currently, human subject federal protections only apply to studies that are funded by federal agencies or clinical investigations that involve products regulated by FDA. The NPRM proposes that regulations pertain to all studies conducted by U.S. institutions, regardless of funding source. As such, clinical studies that were not previously subject to IRB review may require IRB oversight.

To further complicate the issue, in 2016 at the request of Congress, the National Academy of Sciences (NAS) evaluated the effectiveness of the policies surrounding human subjects research and proposed recommendations for improving current regulations [9]. Citing the said problem of regulations lagging behind product development, the NAS made several suggestions, including withdrawing the aforementioned NPRM until several additional independent commissions are appointed to closely examine current regulations.

## Discussion

Clinical investigations are necessary to gather insight about medical treatments, strategies, and devices to determine whether they are safe and effective for use in humans. Investigators are legally required to follow all aspects of federal regulations and guidelines regarding human subject protections.

There are many groups which must be coordinated in order to effectively complete a clinical protocol. These include research sites, sponsors, contract research organizations (CROs), study volunteers, research institutions, and IRBs. IRBs can be valuable sources of information and provide guidance in implementing a clinical study that is in line with current regulations. This is of vital importance for those investigators who wish to conduct their study according to federal regulations, publish study findings, or apply for federal financial support.

At this time, regulations are in the process of being updated in an effort to keep up with the products currently being developed in the rapidly changing health care industry. Many of the proposed NPRM changes could provide clarity for researchers and possibly reduce regulatory burdens; however, other changes such as the biobank requirement to consent would add more regulatory burden to the exploding field of research. As such, an IRB should be consulted early in the study design process to ensure that study documents and procedures are in line with current requirements.

## Conclusions

Researchers must effectively organize their resources to efficiently assess the many issues that arise during a clinical study. There are numerous ethical standards that govern research with human participants in the United States which are codified in the CFR. While regulations may evolve over time, the primary IRB responsibility of protecting the rights and welfare of humans participating in research studies will continue.
